# The Story of the Insane from Year to Year

**Published:** 1901-05-11

**Authors:** 


### THE STORY OF THE INSANE FROM YEAR TO YEAR.

(Thirteenth Series.)

### LANCASHIRE COUNTY ASYLUM, RAINHILL.

On January 1st, 1899, the number in residence was 2,060, and on December 31st it was 2,088. There were 416 first admissions, 31 readmissions, 162 discharged recovered, 22 discharged relieved, and 222 deaths. The average number resident during the year was 2,100, and the total number under treatment was 2,501. Calculated on the admissions of the year the recoveries stand at 36·24 per cent., and calculated on the average number resident the deaths stand at 10·66. Of the year's deaths 93 were caused by cerebral and spinal diseases, 45 by consumption, 11 by pneumonia, 7 by broncho-pneumonia, and 4 by colitis. Of the admissions moral causes, such as domestic trouble, mental anxiety, and religion, are supposed to have caused the insanity in 55 cases, intemperance in drink in 117, "previous attacks" in 80, and hereditary influence in 52. The asylum was so full throughout the year 1899 that it was impossible to meet the demands for accommodation made by the Unions. Dr. Wiglesworth draws attention to the fact that the death rate on the male side was twice as high as it was on the female side, and he shows that this was owing to the number of deaths from general paralysis, of which disease 64 male patients died. He further says that it is "difficult to resist the conclusion that this fell disease is steadily on the increase." We should like to have had Dr. Wiglesworth's opinion as to the cause of this increase, for certainly few men possess greater talents for investigation or have had greater experience with it than he has. Regarding the consumption and the colitis nothing is said. The weekly cost of maintenance was 8s. 3½d. per head.

### THE HEREFORD COUNTY ASYLUM.

Here the numbers were 363 on January 1st, 1899, and 367 on December 31st. There were 50 first admissions, 25 re-admissions, 26 recoveries, 8 discharged relieved, and 34 deaths. The average number resident during 1899 was 367, and the total number under treatment was 435. The recoveries were 41·2 per cent. of the admissions, and the deaths were 9·2 per cent. of the average number resident. Of the 34 deaths, 24 were caused by cerebral and spinal diseases, 4 by consumption, 1 by pneumonia, and 1 by acute enteritis of influenza origin. Moral causes account for the insanity in 4 of the admissions, and physical in 71. Among the latter, intemperance in drink figures in 11 cases, previous attacks in 13, and hereditary influence in 12. The asylum is full, but additions are being made to it, and when these are opened the cases now boarded out at Bow and Beverley asylums will be transferred to Hereford. Dr. Morrison thinks that a standing drawback exists in the definition of insanity by the Poor Law officers, and, as a consequence, cases of senile imbecility are sent to the county asylum who might very well be treated in the workhouses. This is certain; and very slight alterations in the workhouse wards, in the care of the patients and in the diet, would make them admirable places for a large number of so-called lunatics. Dr. Morrison proposes that workhouses should have annexes, known as homes or hospitals for the poor. Unquestionably the word

"workhouse" is a misnomer; but we doubt whether Dr. Morrison's idea will be carried out until the capitation grant to asylum lunatics is withdrawn or also made to workhouse imbeciles; for at present the guardians only pay 5s. a week for patients in the asylum, whereas the inmates of the workhouse cost more than that in Herefordshire.

Dr. Morrison tells us of one of his patients who was fond of swallowing pencils, matches and such things, and who remarked that he had "much sense," but was "not given the sense to use it well." Dr. Morrison thinks that this remark by the lunatic "offers pregnant reflections to those who study social science, and to the criminal anthropologist it supplies a ready explanation for the irrationality and crime which occasionally deface human conduct, with no evidence of malign motive. There also exist germs of a diseased egoism in such a type which readily assimilates the cult of destructive Anarchism, and as parents, become the progenitors of a dangerous and unprofitable class to the community."

This is one of the asylums which is not provided with an isolation hospital, and as there were several cases of erysipelas the need for such an annexe was much felt during the year. When one is built it is to be hoped that it will be of sufficient size to cope with an epidemic, and not one of those useless appendages now run up at many of our county asylums.

It would seem that Dr. Morrison has not yet commenced the training of his attendants and nurses; but he says he should be wanting in the proper apprehension of his duties if he did not recommend that the teaching and training of the attendants be undertaken as soon as the strength of the medical staff will permit; but surely this is one of the things that a medical superintendent might initiate himself, and trust to his committee to give a suitable reward to those of the staff who pass a satisfactory examination. At all events it is much to be desired that Dr. Morrison will inaugurate a three years' course of training; give his own certificate and leave to his staff to please themselves as to the Medico-psychological certificate, which being only a two years' certificate might not be accepted as a passport to membership of nursing institutions.

A ground plan of the additions is given in the report. These additions consist of two blocks, one for 100 women, and the other for 50 men. The position and the mode of attachment to the main building are good. The dormitories are large enough for 41 beds and the beds are placed in four rows; hence the ward is about 35 feet wide (that is about 10 feet more than it ought to be), and perflation of the air must be difficult, the more so as one end of the dormitory is taken up by single bedded rooms and the other by a partition dividing it from the day room. Now, the minimum of fresh air per man is 3,000 cubic feet per hour, and for the insane this is not nearly enough. At this minimum, however, the dormitory must be supplied with 1,230,000 cubic feet of air during the ten hours it is in use. How does Dr. Morrison propose to effect this?

The committee "express their appreciation of the care and ability shown by Dr. Morrison in the management of the Institution." The average weekly cost per head is within a fraction of 9s.

